# Identity Vector Extraction by Perceptual Wavelet Packet Entropy and Convolutional Neural Network for Voice Authentication

**DOI:** 10.3390/e20080600

**Published:** 2018-08-13

**Authors:** Lei Lei, Kun She

**Affiliations:** School of Information and Software Engineering, University of Electrical and Science and Technology of China, Chengdu 610054, China

**Keywords:** i-vector, wavelet entropy, speaker authentication, CNN

## Abstract

Recently, the accuracy of voice authentication system has increased significantly due to the successful application of the identity vector (i-vector) model. This paper proposes a new method for i-vector extraction. In the method, a perceptual wavelet packet transform (PWPT) is designed to convert speech utterances into wavelet entropy feature vectors, and a Convolutional Neural Network (CNN) is designed to estimate the frame posteriors of the wavelet entropy feature vectors. In the end, i-vector is extracted based on those frame posteriors. TIMIT and VoxCeleb speech corpus are used for experiments and the experimental results show that the proposed method can extract appropriate i-vector which reduces the equal error rate (*EER*) and improve the accuracy of voice authentication system in clean and noisy environment.

## 1. Introduction

Speaker modeling technology has been widely used in modern voice authentication for improving accuracy. Among those speaker modeling methods (such as arrange vector, support vector machine (SVM), Gaussian mixture model (GMM) supervector, joint factor analysis (JFA) and so on), i-vector model has wide applicability, because it is easy to implement and gives good performance [1]. Over the recent decades, the i-vector model has become a reliable and fast speaker modeling technology for voice authentication in a wide range of applications such as access control and forensics [2,3].

Speech utterance contains a huge number of redundancies. Thus, for i-vector extraction, it should be converted into feature vectors where the valuable information is emphasized and redundancies are suppressed. Mel-frequency cepstral coefficient (MFCC) is commonly used spectral features for speech representation. Although MFCC achieved great success in early speech representation, its disadvantage is to use short-time Fourier transform (SFT), which has weak time-frequency resolution and an assumption that the speech signal is stationary. Therefore, it is relatively hard to represent the non-stationary speech segment (such as plosive phonemes) by the MFCC [4].

Wavelet increasingly becomes an alternative to Fourier transform due to its multi-scale resolution which is suitable for analyzing non-stationary signal. Over recent years, many wavelet-based spectral features such as wavelet-based MFCC [5], wavelet-based linear prediction cepstral coefficient (LPCC) [4], wavelet energy [6] and wavelet entropy [7] have been proposed by researchers. Among those wavelet-based features, wavelet entropy has some superior features. Wavelet entropy is sensitive to singular point of signal, so it can highlight the valuable information of speech signal [8]. Moreover, it has ability to significantly reduce the size of data, which is helpful for speeding up back-end speaker modeling and classification process [9].

Typically, wavelet entropy feature extraction is based on wavelet transform (WT) or wavelet packet transform (WPT). However, WT cannot provide high enough high-frequency resolution due to the fact that WT just decomposes low-frequency part of signal. Although WPT, which performs decomposition on both low- and high-frequency part of signal, provides richer analysis than WT, but the time required to implement WPT will become very heavy as the increasing of the its decomposition level [4]. Currently, a case of WPT with irregular decomposition, named perceptual wavelet packet transform (PWPT), is proposed for speech enhancement [10]. The main advantage of PWPT is that it, like WPT, can provide rich analysis but its time cost is much lower than WPT due to the irregular decomposition. Moreover, it simulates the human auditory system to perceive the frequency information of speech, which is helpful for analyzing speech information and suppressing speech noise [10,11]. Therefore, PWPT seems to be effective for extracting robust wavelet entropy feature vector.

Once a speech utterance is converted into a set of feature vectors, the i-vector can be extracted based on those feature vectors. A key issue of i-vector extraction is how to estimate the frame posteriors of a feature vector. For standard i-vector extraction [12], the frame posteriors are estimated using Gaussian mixture model (GMM). However, inspired by the success of deep learning in speech recognition, researchers trend to replay the GMM by deep model. Actually, phonetic deep neural network (DNN) has been used instead of GMM to estimate the frame posteriors and often gives more reliable frame posterior than GMM in several works [13,14,15]. Convolutional neural network (CNN) is other type of deep model and has been proven to be better than DNN in speech recognition cases [16,17]. Thus, CNN may be a good choice to estimate reliable frame posteriors for i-vector extraction.

In this paper, many i-vector extraction methods are investigated and a new method for i-vector extraction is proposed. The main works of this paper are summarized as follows:

(1) Design a PWPT according to the human auditory model named Greenwood scale function.
(2) Utilize the PWPT to convert speech utterance into wavelet entropy feature vectors.
(3) Design a CNN according to the phonetic DNN.
(4) Utilize the CNN to estimate frame posteriors of feature vector from i-vector extraction.

The rest of paper is organized as follows: Section 2 discusses how to extract the wavelet entropy feature from speech utterance. Section 3 discusses the i-vector extraction method. Section 4 describes voice authentication task used for performance evaluation, and Section 5 reports the result of experiments. Finally, a conclusion is given out in Section 6.

## 2. Wavelet Entropy Feature Extraction

### 2.1. Wavelet Packet Transform

As its name shows, wavelet entropy is based on wavelet analysis. Thus, our description starts with the Wavelet Packet Transform (WPT).

WPT is a wavelet analysis method. It is widely used in various scientific and engineering fields such as speech processing, image processing, security system, biomedicine and so on. In practice, WPT is implemented by two recursive band-pass filtering processes which are defined as:

(1) $$\{\begin{array}{c} w_{j+1}^{2p}\left(l\right)=\sum_{k}h\left(k-2l\right)w_{j}^{p}\left(k\right)\\ w_{j+1}^{2p+1}\left(l\right)=\sum_{k}g\left(k-2l\right)w_{j}^{p}\left(k\right)\\ w_{0}^{0}\left(l\right)=x\left(l\right) \end{array};p=0,\;1,\;2,\;\ldots ,\;2^{j};j=1,\;2,\;3,\;\ldots ,\;J$$

where x(l) is a signal to be decomposed and J is the maximum decomposition level of WPT. h(·) and g(·) are the couple of low-pass and high-pass filters, which are constructed by a mother wavelet and the corresponding scale function. $w_{j}^{p}\left(\cdot \right)$ is the *p*-th WPT sub signal at level *j*. The $w_{j+1}^{2p}\left(\cdot \right)$ is the low-frequency of $w_{j}^{p}\left(\cdot \right)$, and $w_{j+1}^{2p+1}\left(\cdot \right)$ is the high-frequency part of $w_{j}^{p}\left(\cdot \right)$.

WPT regularly decomposes both the low-frequency and high-frequency parts of signals, so it provides rich time-frequency analysis as usual. However, the computational cost of WPT will become very high due to the regular decomposition.

### 2.2. Perceptual Wavelet Packet Transform

Perceptual wavelet packet transform (PWPT) is a case of WPT with irregular decomposition. The key issue for PWPT is how to design its decomposition process to adopt a given signal. For speech signal, the PWPT is usually designed to simulate human auditory perception process [10].

This paper designs a PWPT which simulates a auditory perception model named Greenwood scale frequency function (GSFF). This human auditory model is proposed by Greenwood in [18] and shows that mammals perceive sound frequency on a logarithmic scale along the cochlea, which corresponds to a non-uniform frequency resolution. The GSFF is defined by:

(2) $$f\left(x\right)=A\left(10^{\alpha x}-k\right)$$

where f(x) is the perceived frequency and x is the normalized cochlea position with a value of from zero to one. k, A, α are species-dependent constants. The work in [19] shows k can be estimated as 0.88 for mammal and A, α are defined by:

(3) $$A=\frac{f_{\min}}{1-k}$$

(4) $$\alpha =\log_{10}\left(\frac{f_{\max}}{A}+1\right)$$

where the $f_{\min}$ and $f_{\max}$ are determined by auditory frequency range of a species. For human, $f_{\min}=20\;\mathrm{Hz}$ and $f_{\max}=20\;\mathrm{kHz}$.

**Figure 1:**
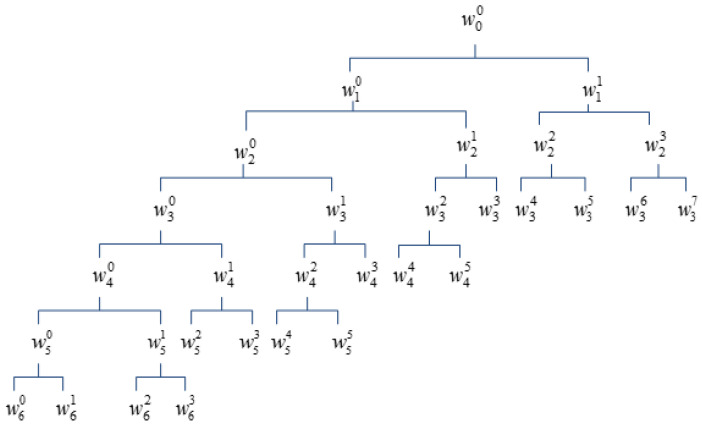
The decomposition structure of PWPT.

Using human-specific GSFF, this paper gets 24 perceived frequencies whose positions are linearly spaced along the cochlea. The useful speech frequency is from 300 Hz to 3400 Hz in phony, so only the first 16 received frequencies are used to design the PWPT. Figure 1 shows the decomposition structure of the PWPT.

**Figure 2:**
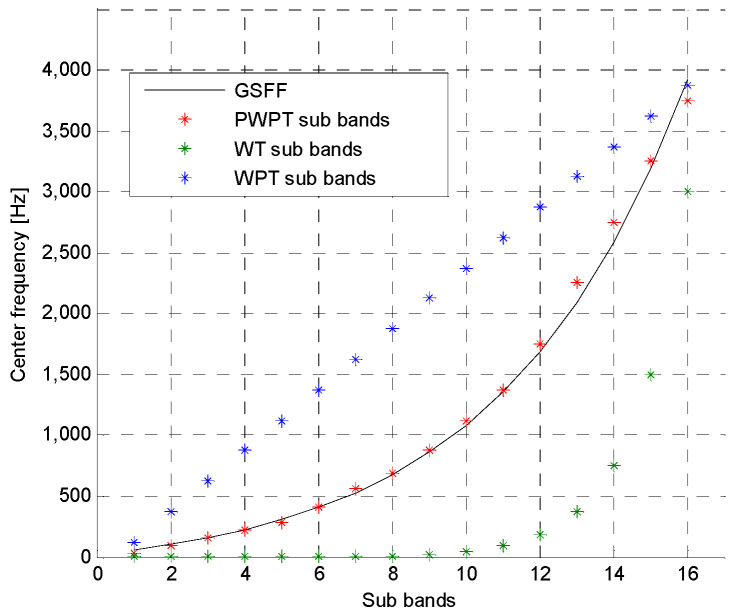
Comparison of PWPT, WT and WPT.

In the figure, the $w_{0}^{0}$ represents a speech segment to be analyzed. The terminal nodes of the tree represent 16 PWPT sub signals corresponding to 16 sub bands whose center frequencies approximate the 16 perceived frequencies. Figure 2 shows comparison of PWPT, WT and WPT.

In the figure, the PWPT can very closely approximate the human auditory perception model compared with WT and WPT. 

Usually, PWPT offers some useful properties for feature extraction. Firstly, PWPT provides high resolution for valuable voice information and low resolution for the redundancies [20], which gives out expectable analysis result. Secondly, the perceptual decomposition process of PWPT is very useful for suppressing speech noise [11], so it is possible to build anti-noise spectral feature procedure based on PWPT. Thirdly, the computational cost of PWPT is not very heavy due to the irregular decomposition.

### 2.3. PWPT-Based Wavelet Entropy Feature

To accurately represent the speech information, this paper converts speech utterance into wavelet entropy feature based on the above PWPT.

At the start of the wavelet entropy feature extraction, speech utterance is processed by a per-processing procedure which consists of three sequential stages: normalization, framing and silence removing. Through normalization, the effect of volume is discard and utterance becomes comparable. Assume a digital speech utterance denoted by {x[i]}(i=1, 2, 3, …, I) where x[i] is the sampling point in the speech utterance, then the normalization is defined as:

(5) $$x_{n}\left[i\right]=\frac{x\left[i\right]-m}{\sigma };\;i=1,\;2,\;3,\;\ldots ,\;I$$

where $x_{n}$ is the normalized utterance. I<+∞ is the length of the speech utterance x, m and σ are the mean and standard deviation of the x. In framing process, the normalized utterance $x_{n}$ is divided into many short-term frames. Each frame in this paper contains 512 sampling points because the 512 points contain enough information for feature extraction and the change in them is not too much [21]. In silence removing stage, the silence frames (whose energies are less than a threshold) are discard and the active frames (whose energies are greater than threshold) are remained.

After pre-processing procedure, the speech utterance is divided into a frame set which contains *N* active frames. PWPT decomposes each active frame into 16 sub frames (signals), designated by $\left\{w_{1},\;w_{2},\;\ldots ,\;w_{16}\right\}$. To suppress ambient noise in sub frame, a de-noising process [11] is used on the each sub frame. The de-noising process is defined as:

(6) $$d\left[i\right]=\{\begin{array}{cc} w\left[i\right], & \left|w\left[i\right]\right|>T\\ 0, & \left|w\left[i\right]\right|\leq T \end{array};\;i=1,\;2,\;3,\;\ldots ,\;I$$

where I is the length of the sub frame w. d is de-noised sub frame. T is a threshold and is defined by:

(7) $$T=\frac{M\left(w\right)}{C}\sqrt{2\ln\left(I\right)},$$

where M(w) is the median absolute deviation estimation of the w. C is empirical constant and is usually set to 0.675 for ambient noise [11].

The wavelet entropy is calculated based on the ${\left|d\left[i\right]\right|}^{2}$. This paper calculated four commonly used entropies which are defined as follows:

Shannon entropy:

(8) $$\begin{array}{l} H\left(d\right)=-\sum_{i=1}^{I}{\left|p_{i}\right|}^{2}\log\left({\left|p_{i}\right|}^{2}\right),\\ p_{i}=\frac{{\left|d\left[i\right]\right|}^{2}}{\sum_{j=1}^{I}{\left|d\left[j\right]\right|}^{2}} \end{array}$$

Non-normalized Shannon entropy:

(9) $$H\left(d\right)=-\sum_{i=1}^{I}{\left|d[i]\right|}^{2}\log\left({\left|d[i]\right|}^{2}\right)$$

Log-energy entropy:

(10) $$H\left(d\right)=\sum_{i=1}^{I}\log{\left|d[i]\right|}^{2}$$

Sure Entropy:

(11) $$H\left(d\right)=\sum_{i=1}^{I}\min(\left|d\right|{\left[i\right]}^{2},\varepsilon^{2});\;\varepsilon =2\;\mathrm{as}\;\mathrm{usual}$$

According to the above calculation, an active frame can be transformed into a feature vector denoted by $v={\left[H\left(d_{1}\right),\;H\left(d_{2}\right),\;\ldots ,\;H\left(d_{16}\right)\right]}^{T}$ where v is called PWE vector in this paper. Therefore, speech utterance which contains *N* active frames is mapped into a set of PWE vectors denoted as:

(12) $$U=\left\{v_{1},\;v_{2},\;\ldots ,\;v_{N}\right\}$$

## 3. i-Vector Extraction

### 3.1. i-Vector Definition and Extraction Framwork

In i-vector theory, feature vector $v_{t}$ of a speech utterance is assumed to be generated by the following distribution:

(13) $$v_{t}\sim \sum_{k=1}^{L}\alpha_{tk}N\left(u_{k}+T_{k}\omega ,\Sigma_{k}\right);$$

where the N(⋅) is a normal distribution, and $u_{k},\Sigma_{k}$ are its mean and covariance. $T_{k}$ is a matrix and represents a low-rank subspace called total variability subspace. $\alpha_{tk}$ is the *k*-th frame posterior of $v_{t}$ in a universal background model (UBM). L is the number of frame posteriors of the feature vector $v_{t}$ and is equal to 2048 in typical i-vector extraction methods. ω is a utterance-specific standard normal-distributed latent vector and its maximum posterior point (MAP) estimation is defined as i-vector.

**Figure 3:**
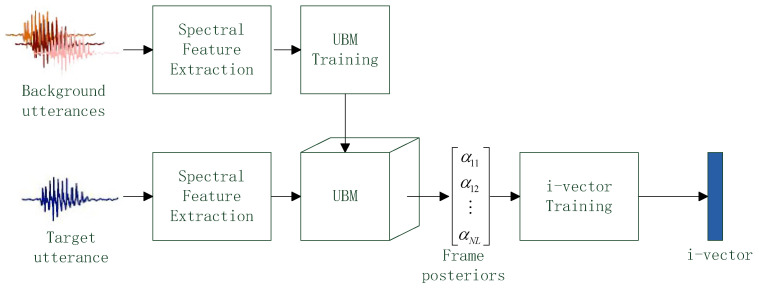
The i-vector extraction framework.

Based on the above assumption, the standard i-vector extraction framework is proposed in [12]. The framework is shown in Figure 3.

There are two types of speech utterances. The background utterances contain thousands of speech samples spoken by lots of persons and the target utterance comes from a given speaker and the purpose of i-vector extraction is convert target utterance into a i-vector. In the framework, all speech utterances are converted into spectral feature vectors. UBM is trained by the feature vectors from background utterances and L frame posteriors of a feature vector from the target utterance are estimated based on the trained UBM. Finally, through the i-vector training procedure described in [22], i-vector is generated based on the frame posteriors. One i-vector corresponds to one target utterance, and the dimension of i-vector is 300~400 as usual.

### 3.2. Typical i-Vector Extraction

The key issue of i-vector extraction is how to implement UBM to estimate the frame posterior. In the standard i-vector, UBM is implemented by a Gaussian mixture model (GMM) which contains *L* weighted Gaussian functions. Assume a target utterance is represented by a set of feature vectors $\left\{v_{1},\;v_{2},\;\ldots ,\;v_{N}\right\}$. The *k*-th frame posterior $\alpha_{tk}$ of the feature vector $v_{t}$ is calculated by:

(14) $$\alpha_{tk}=\frac{\pi_{k}G_{k}\left(v_{t}\right)}{\sum_{i=1}^{L}\pi_{i}G_{i}\left(v_{t}\right)}$$

where $\pi_{i}G_{i}\left(\cdot \right)$ is the *i*-th weighted Gaussian function of the GMM.

Over the last decade, GMM is the state-of-art work for the frame posterior estimation. However, GMM just considers the inner information within feature vector and is trained in generative way, so it cannot generate reliable frame posteriors [13]. Moreover, in standard i-vector extraction, speech utterances are represented by MFCC feature vectors which are not very powerful for speech representation.

**Figure 4:**
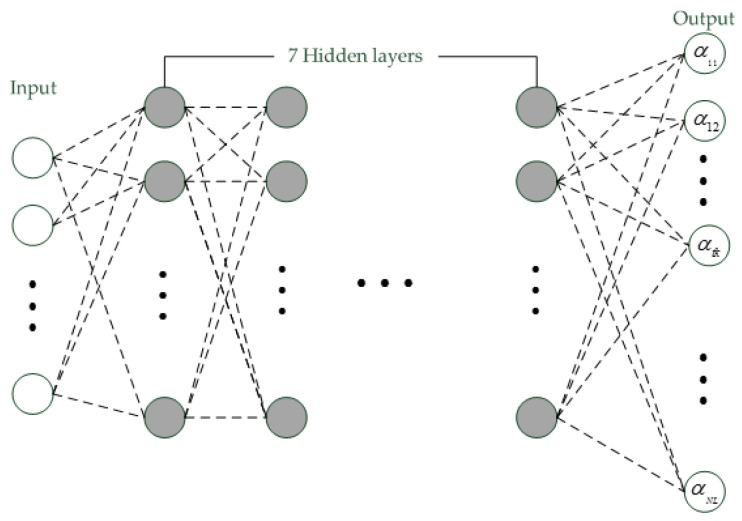
The structure of DNN.

The success of deep learning in speech recognition motivates researchers to use DNN to estimate the frame posterior. Compared with GMM, DNN considers the inner information within feature vector and context information between feature vectors together and is discriminatively trained. Thus, it often generates more reliable frame posteriors than GMM [14]. The typical deep structure used for posterior estimation is the phonetic DNN, which is shown in Figure 4.

This DNN contains nine full-connected layers with sigmoid activation. The input layer is a stacked set of 11 feature vectors. If feature vector is hx1 vector, then the input layer is 11 hx1 vector. There are seven hidden layers in the DNN, and each hidden layer contains 1024 nodes. The output layer contains 2048 nodes and each node represents a frame posterior. Like GMM, this DNN is also trained by the feature vectors of background utterances. Assume the input layer is $V_{t}$, then the frame posterior $\alpha_{tk}$ is represented by the *k*-th node of output layer in the DNN.

Although this DNN can give more reliable frame posteriors than GMM, but its huge number of parameters also improves the computational complexity and storage cost. Moreover, the speech utterances in this i-vector extraction are also represented by MFCC feature vectors.

### 3.3. i-Vector Extraction with CNN

**Figure 5:**
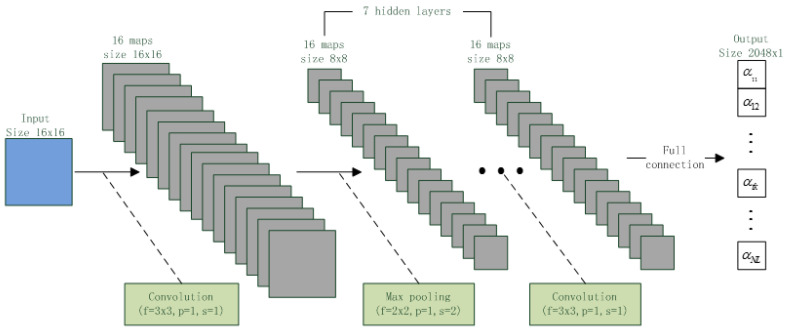
CNN structure.

CNN is new type of deep model proposed in few two years. Due to the convolution connection between adjacent layers, the CNN has much smaller parameter size than DNN, which speeds up the CNN computation process. Moreover, in recent image and speech works, CNN is often found to outperform DNN and be noise-robust [16]. This motivates us to design a CNN to implement UBM. The structure of the designed CNN is shown in Figure 5.

In the figure, green blocks show connection operators between adjacent layers, where the f, p, s represents the filter size, padding size and stride size, respectively. This CNN has 10 layers with ReLU activation. The input layer of the CNN is a 16 × 16 matrix which is formed by 16 16 × 1 feature vectors. There are seven hidden layers and each layer contains 16 8 × 8 feature maps. The output layer contains 2048 nodes and fully connects to the last hidden layer. Table 1 shows the difference between the CNN and DNN.

As the table shown, the node size of the DNN and CNN are same, but the CNN has much less parameters than the DNN.

**Figure 6:**
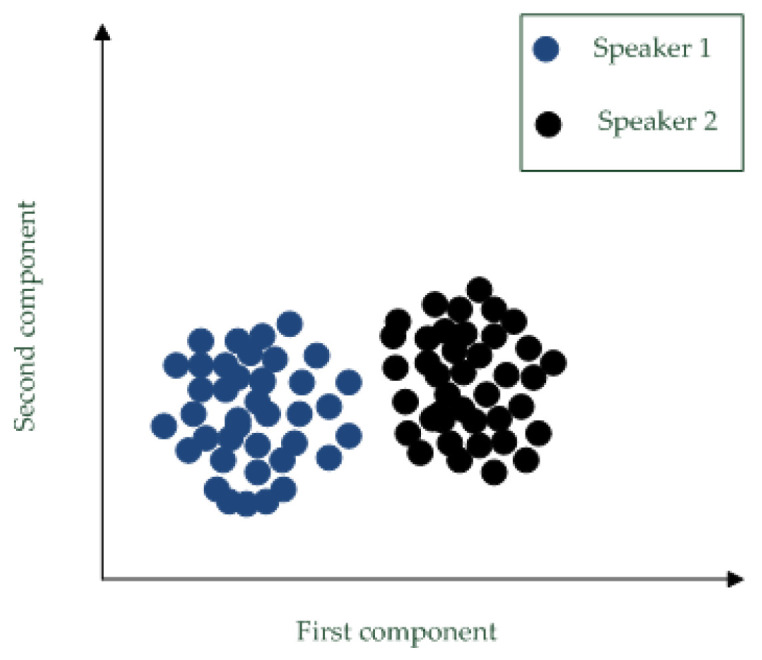
I-vectors for two speakers.

In the proposed i-vector extraction method, the speech utterances are represented by wavelet packet entropy (WPE) feature vectors, and the CNN is used to implement UBM. For i-vector extraction, the CNN is trained by feature vectors of background utterances. Assume the input matrix is $V_{t}$, then the frame posterior $\alpha_{tk}$ is represented by the *k*-th node of output layer in the CNN. Figure 6 shows the i-vectors for two speakers. Each speaker provides 40 speech utterances and one utterance corresponds to one i-vector extracted by the proposed method. To show those i-vectors, principle component analysis (PCA) maps the i-vectors into 2D points. This figure shown that the extracted i-vectors are discriminative for different individuals.

## 4. Voice Authentication

**Figure 7:**
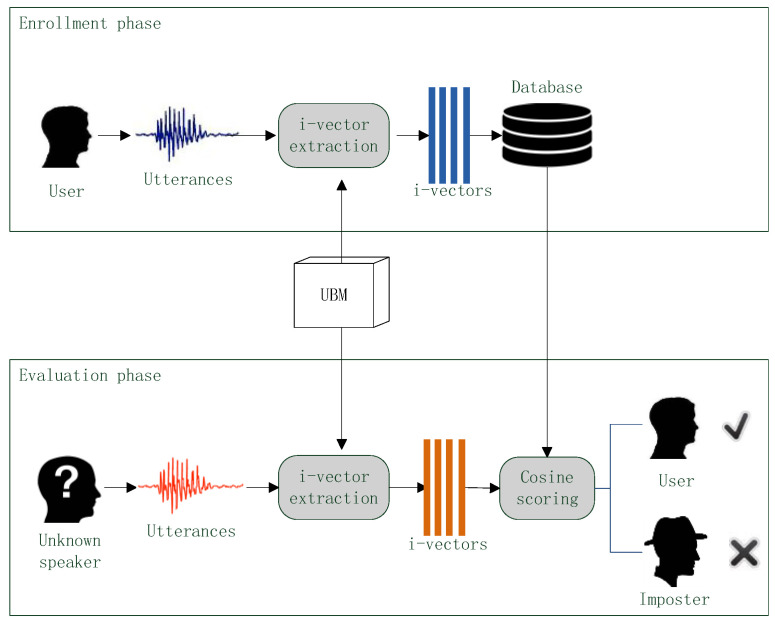
The flow chart of voice authentication process.

In the experiments of this paper, different i-vector extraction methods with different spectral features are used for voice authentication, and their performances are evaluated according to the authentication results. The flow chart of the voice authentication is shown in Figure 7.

In the voice authentication sense, there are three types of speakers: user, imposter and unknown speaker. User is correct speaker which the voice authentication system should accept, imposter is adverse speaker who should be rejected by the system and Unknown speaker should be verified by the system.

A voice authentication can be divided into two phases: enrollment and evaluation. In the enrollment phase, user provides one or more speech utterances. An i-vector extraction method converts those speech samples into i-vectors and then those i-vector are stored in a database. In the evaluation phase, an unknown speaker also provides one or more speech samples. The extraction method converts these samples into i-vectors as well and then a scoring method compares the i-vectors of unknown speaker against the i-vectors in database to produce verification score. If the score is less than a given discrimination threshold, the unknown speaker is considered as the user and the authentication result is acceptance; if the score is greater than the threshold, the unknown speaker is considered as a imposter and the authentication result is rejection.

In the voice authentication, the UBM is trained beforehand and is used in both of enrollment and evaluation phrase for i-vector extraction. To better verify the quality of different i-vector extraction methods, the scoring method should be simple [23]. Thus, the cosine scoring (CS) [24] is used.

## 5. Results and Discussion

### 5.1. Database and Experimental Platform and Performance Standards

In this paper, the TIMIT [25] and Voxceleb [26] speech corpus are used for experiments. The TIMIT corpus contained speech data from 630 English speakers. In TIMIT, each speaker supplied 10 speech utterances and each utterance lasted 5 s. All speech utterances of TIMIT were recorded by microphone in a clean lab environment and the sampling rate of all utterances is 16 KHz. The Voxceleb dataset contained 153,516 speech utterances of 1251 English speakers. In Voxceleb, Each speakers provided 45~250 utterances in average and speech duration ranged from 4 s to 145 s. All speech utterances in Voxceleb were recorded in the Wild at 16 Hz sampling rate. In this paper, clean speech data came from TIMIT and noisy speech data came from Voxceleb.

Experiments in this section simulated voice authentication task and were implemented by MATLAB 2012b (MathWorks, Natick, USA) which was carried on a computer with i5 CPU and 4 GB memory. To quantitatively analyze the performance of different i-vector extraction methods, two performance standards were used. The first one was accuracy, which was the typical performance standard and was defined by the sum of true rejection rate and true acceptance rate. Another one is equal error rate (*EER*), which was a performance standard suggested by National Institute of Standards and Technology (NIST). It was defined as the equal point of false rejection rate and false acceptance rate. This standard represented the error cost of a voice authentication system, and low *EER* corresponds to good performance.

### 5.2. Mother Wavelet Selelction

This section tested different mother wavelets to find the optimum one for the PWPT. According to the Daubechies theory [27],the wavelets in Daubechies and Symlet families were useful because they had the smallest support set for given number of vanish moments. In this experiment, 10 Daubechies wavelets and 10 Symlet wavelets, which were denoted by db 1~10 and sym 1~10, were tested. 3000 speech utterances were randomly selected from the TIMIT and Voxceleb and all utterances were decomposed by the proposed PWPT with different mother wavelets. Energy-to-Shannon entropy ratio (*ESER*) was used performance standard of the above mother wavelets and was defined by:

(15) $$ESER=\sum_{n=1}^{16}\frac{E_{n}}{H_{n}}$$

where $E_{n}$ was the energy of the nth PWPT sub signal, and $H_{n}$ was the Shannon entropy of the sub signal. *ESER* measured the analysis ability of a mother wavelet and high *ESER* corresponded to good-performance mother wavelet [28]. The experiment result was shown in Table 2.

In the table, the db 4 and sym 6 obtained the highest *ESER*. Thus, the db 4 and sym 6 were good mother wavelets for PWPT. However, sym 6 was a complex wavelet whose imaginary transform cost extra time, so the computational complexity of sym 6 was higher than db 4. Thus, db 4 was the optimum mother wavelet.

### 5.3. Evaluation of Different Spectral Featrures

This section studied the performance of different spectral features. Four types of entropy features such as Shannon entropy (ShE) non-normalized Shannon entropy (NE), log-energy entropy (LE) and sure entropy (SE), and two typical spectral features such as MFCC and LPCC were tested. The proposed CNN was used as UBM which was trained by all of speech utterances in TIMIT and Voxceleb.

The first experiment analyzed the performance of four wavelet entropies. WT, WPT and PWPT were used for wavelet entropy feature extraction. 6300 speech utterances of 630 speakers in TIMIT were used for this experiment. The experiment result was shown in Table 3.

In the Table, all of WT-based entropies obtained the highest *EER*, which shown that WT might not be effective for speech feature extraction. One reason of this was the WT had low resolution for high-frequency speech which may contains valuable detail information of signal. The ShE and NE with WPT and PWPT obtained low *EER*s, which shown that the WPT- and PWPT-based ShE and NE were good feature for speech representation. This was because the ShE and NE were more discriminative than other entropies [29]. Although both of the two feature had good performance for speech representation, but NE was fast to be computed compared with ShE.

**Figure 8:**
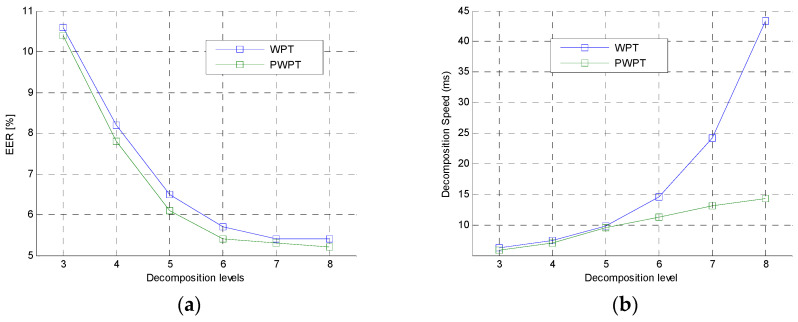
Comparison of WPT and PWPT in feature extraction. (**a**) *EER*s of WPT and PWPT. (**b**) Time cost of WPT and PWPT.

The second experiment was to further analyze the performance of the WPT and PWPT in feature extraction. In this experiment, PWPT and WPT with different decomposition levels were used to extract NE from speech utterance. The 6300 TIMIT speech utterances were also used in this experiment. Comparison of PWPT and WPT was shown in Figure 8.

In the figures, the *EER* curve of WPT was very close to the *EER* curve of PWPT. This shown that the typical WPT and the PWPT had same analysis performance in general. However, the time cost of WPT was much higher than the time cost of PWPT when the decomposition level was greater than 4, which shown that PWPT was a faster tool than WPT. This was because PWPT irregularly decomposed speech signal while the WPT performed a regular decomposition on signal.

The last experiment in section is to compare the performance of the waveket-based NEs (PWPT-NE, WPT-NE and WT-NE) with typical MFCC and LPCC features in clean and noisy environment. The 6300 clean speech utterances of 630 speakers in TIMIT and 25,020 noisy speech utterances of 1251 speakers in Voxceleb were used for this experiment. The wavelet entropies were calculated on wavelet power spectrum, and MFCC and LPCC were calculated on the Fourier power spectrum. The experimental result was shown in Table 4.

In the tale, *EER*s of MFCC and LPCC were higher than the *EER* of wavelet-NEs and their accuracies were lower than wavelet-NE’s, which shown that the wavelet-NEs had better performance than the MFCC or LPCC. One reason of this was the wavelet which has richer time-frequency resolution than Fourier transform for analyzing the non-stationary speech segments. For noisy speech, all *EER*s were increased and all accuracies were decreased, because the noise could lead to performance degradation. However, PWPT-NE still got better performance than other. The reason of this was the perception decomposition of PWPT simulated human auditory perception process to suppress the noise in speech but other transforms could not do that.

### 5.4. Evaluationof Different UBMs

This experiment investigated the performance of different UBMs. GMM with 1024 mixtures, GMM with 2048 mixtures, GMM with 3072 mixtures, DNN and CNN were compared and the PWPT-NE was used as spectral feature. All UBMs were trained by the all speech utterances of TIMIT and Voxceleb.

The first experiment was to compared the three UBMs in clean and noisy environment. As the above experiment did, the 6300 clean speech utterances in TIMIT and 25,020 noisy speech utterances in Voxceleb were used for this experiment. The experimental result was shown in Table 5.

In the table, the GMMs obtained the low accuracy and high *EER*, which shown that the GMMs had bad performance compared with the deep models. The reason of this had shown in [13]. Furthermore, the DNN and CNN had same *EER*s and accuracies in general for clean speech, but the DNN got higher *EER* and lower accuracy than CNN for noisy speech, which shown the CNN’s superiority in resisting noise. In fact, CNN had been exported to be noise-robust in speech recognition [30].

**Figure 9:**
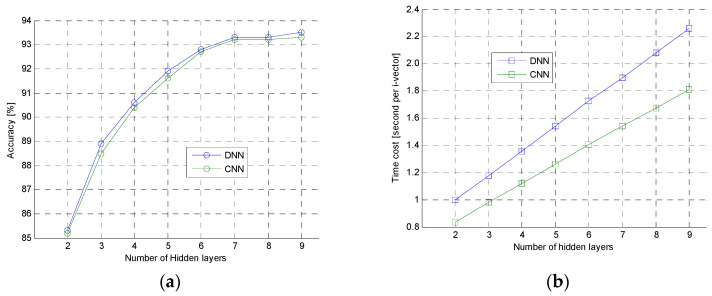
The accuracy and computational speed of CNN and DNN. (**a**) Accuracy (**b**) Computational speed.

The second experiment was to further analyze the performance of DNN and CNN. In this experiment, the 6300 clean speech samples were used to test DNN and CNN with different hidden layers. The experimental result was shown in Figure 9. In the Figure 9a, the accuracy curve of DNN and CNN were very close, but, in the Figure 9b, computational speed of DNN was slower than the CNN when they had same hidden layers. Those shown that the proposed CNN had same ability as the typical DNN, but the speed of CNN was faster than the DNN. This was because the CNN had much less parameters which should be computed for i-vector extraction than DNN, and activation function of CNN was ReLU, which was simpler and faster than activation function of sigmoid used in DNN.

### 5.5. Comparison of Different i-Vector Extraction Methods

This section compared six different i-vector extraction methods such as MFCC + GMM [12], WPE + GMM, WPE + DNN, MFCC + DNN [13], MFCC + CNN and WPE + CNN. The 6300 clean and 25,020 noisy speech utterances were used for this experiment. The experimental result was shown in Table 6.

In the table, the GMM-based methods obtained the highest *EER* and the lowest accuracy. This shown that the deep-based methods had better ability to extract robust i-vector than the GMM-based methods. The WPE + CNN obtained the lowest *EER* and higher accuracy, which shown the proposed model was good at extracting appropriate i-vector for voice authentication. On the other hand, for noisy speech, the performance of MFCC-based methods dropped rapidly, but the performance of WPE-based methods almost had little change. The probable reason of this was that the both of PWPT had noise-suppression ability but Fourier transform did not have.

**Figure 10:**
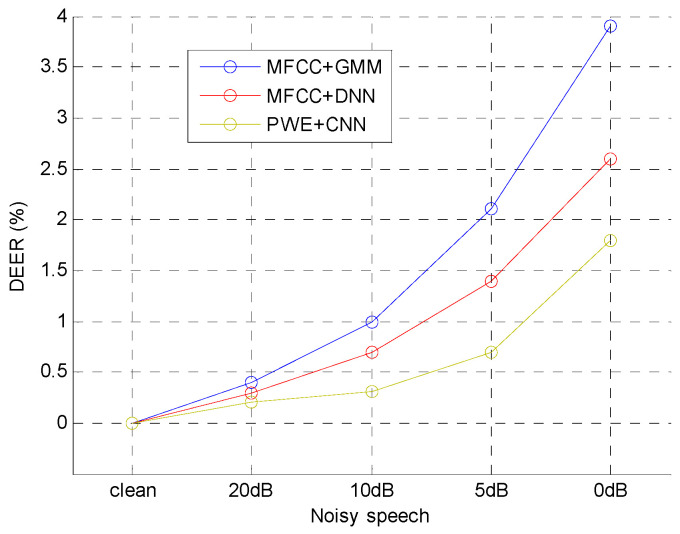
*DEER*s of the three i-vector extraction methods.

The second experiment is to test the robustness of the typical methods and the proposed method in noisy environment. Four types of additive Gaussian white noises (AGWN) generated by MATLAB function were added into the 6300 clean speech utterances in TIMIT. The signal-to-noise ratio (SNR) of noisy speech utterances were 20 dB, 10 dB, 5 dB and 0 dB, and the noisy strength of those speech utterances were 20 dB < 10 dB < 5 dB < 0 dB. The performance standard was delta value of *EER* (*DEER*) which was defined as:

(16) $$DEER=\left(EER_{n}-EER_{0}\right)$$

where $EER_{n}$ was the *EER* for noisy speech and $EER_{0}$ was *EER* for clean speech. The experimental result was shown in Figure 10.

In the figure, *DEER*s of all methods were increased by less than 1% for 10 dB noisy speech, which shown all of methods had ability to resist weak noise. For 0 dB noisy speech, the *DEER*s of MFCC + GMM and MFCC + DNN increased more than 2.5%, but the *DEER* of PWE + CNN increased less than 2%, which shown that the PWE was more robust than the other two methods in noisy environment.

## 6. Conclusions

This paper proposes a new method for i-vector extraction. In the method, a designed PWPT simulate human auditory model to perceptively decompose speech signal into 16 sub signals, and then wavelet entropy feature vectors are calculated on those sub signals. For i-vector extraction, a CNN is designed to estimate the frame posteriors of the wavelet entropy feature vectors.

The speech utterances in TIMIT and Voxceleb are used as experimental data to evaluate different methods. The experimental result shown that the proposed WPE and CNN had good performance and the WPE + CNN method can extract robust i-vector for clean and noisy speech.

In the future, the study will focus on new speech feature and the perceptual wavelet packet algorithm. On the one hand, the perceptual wavelet packet will be implemented by parallel algorithm for reducing the computational expense. On the other hand, the new features, such as combination of multiple entropies, will be tested for further improving the speech feature extraction.

## Figures and Tables

**Table 1:** The Comparison of the DNN and CNN.

Layer	Shape		Node Size		Parameter Size	
	DNN	CNN	DNN	CNN	DNN	CNN
Input Layer	256 × 1, 1	16 × 16, 1	256	256	226,144	272
Hidden Layer 1~7	1024 × 1, 1	8 × 8, 16	1024	1024	1,048,576	160
Output Layer	2048 × 1, 1	2048 × 1, 1	2048	2048	131,072	131,072

**Table 2:** *ESER* of PWPT with different mother wavelet.

Wavelets	*ESER*s	Wavelets	*ESER*s	Wavelets	*ESER*s	Wavelets	*ESER*s
Db 1	888.37	Db 6	896.53	Sym 1	888.35	Sym 6	908.39
Db 2	890.32	Db 7	891.69	Sym 2	890.36	Sym 7	902.44
Db 3	897.44	Db 8	890.84	Sym 3	894.93	Sym 8	898.37
Db 4	907.45	Db 9	888.21	Sym 4	899.75	Sym 9	896.35
Db 5	901.41	Db 10	884.50	Sym 5	903.82	Sym 10	891.34

**Table 3:** *EER* (%) of recognition system with different wavelet entropy features.

	WT	WPT	PWPT
ShE	8.51	5.46	5.49
NE	8.57	5.53	5.51
LE	9.03	6.67	6.78
SE	8.91	6.23	6.27

**Table 4:** *EER* and accuracy of spectral features.

Spectral Features	*EER* (%)		Accuracy (%)	
	Noisy	Clean	Noisy	Clean
PWPT-NE	6.24	5.53	90.13	92.14
WPT-NE	7.11	5.51	89.47	92.48
WT-NE	10.27	8.43	86.39.	90.12
MFCC	11.43	9.23	83.10	89.31
LPCC	11.77	9.31	83.24	88.97

**Table 5:** The comparison of three UBMs.

UBMs	*EER* (%)		Accuracy (%)	
	Noisy	Clean	Noisy	Clean
GMM (1024)	13.42	11.96	82.75	86.19
GMM (2048)	11.19	9.23	86.17	89.94
GMM (3072)	9.78	7.54	88.73	91.97
DNN	7.11	5.51	89.47	92.48
CNN	6.24	5.53	90.13	92.14

**Table 6:** The performance of i-vector extraction methods.

Strategies	EER (%)		Accuracy (%)	
	Noisy	Clean	Noisy	Clean
MFCC + GMM	13.02	9.15	80.74	89.59
WPE + GMM	13.17	10.97.	85.97	87.49
MFCC + DNN	10.15	5.68	85.6	91.91
WPE + DNN	8.76	6.87	90.17	92.87
MFCC + CNN	8.02	5.97	86.43	91.48.
WPE + CNN	6.24	5.53	90.13	92.14

## References

[1] Kenny P., Puellet P., Dehak N. (2008). A study of inter-speaker variability in speaker verification. Audio Speech Lang. Process..

[2] Sizov A., Khoury E., Kinnunen T. (2016). Joint speaker verification and antispoofing in the i-vector space. IEEE Trans. Inf. Forensics Secur..

[3] Yu C., Zhang C., Kelly F., Sangwan A., Hansen J.H. Text-available speaker recognition system for forensic applications. Proceedings of the Interspeech.

[4] Daqrouq K., Azzawi K.A. (2012). Average framing linear prediction coding with wavelet transform for text-independent speaker identification system. Comput. Electr. Eng..

[5] Srivastava S., Bhardwaj S., Bhandari A., Gupta K., Bahl H., Gupta J.R.P. (2013). Wavelet packet based Mel frequency cepstral coefficient features for text independent speaker identification. Intell. Inf..

[6] Wu X.Q., Wang K.Q., Zhang D. (2005). Wavelet Energy Feature Extraction and Matching for Palm print Recognition. J. Comput. Sci. Technol..

[7] Jiao M., Lou L., Geng X. Speech enhancement based on the wiener filter and wavelet entropy. Proceedings of the International Conference on Fuzzy Systems and knowledge Discovery.

[8] Besbes S., Lachiri Z. Wavelet packet energy and entropy features for classification of stressed speech. Proceedings of the 17th International Conference on Sciences and Techniques of Automatic Control and Computer Engineering.

[9] Daqrouq K., Sweidan H., Balamesh A., Ajour M.N. (2017). Off-line handwritten signature recognition by wavelet entropy and neural network. Entropy.

[10] Dachasilaruk S., Bleeck S., White P. Improving speech intelligibility in perceptual wavelet packet-based speech coding for cochlear implants. Proceedings of the International Conference on Biomedical Engineering and Informatics.

[11] Chen F., Li C., An Q., Liang F., Qi F., Li S., Wang J. (2016). Noise suppression in 94 GHz Radar-detected speech based on perceptual wavelet packet. Entropy.

[12] Dehak N., Kenny P.J., Dehak R. (2011). Front-end factor analysis for speaekr verification. IEEE Trans. Audio Speech Lang. Process..

[13] Lei Y., Scheffer N., Ferer L., McLaren M. A novel scheme for speaker recognition using a phonetically-aware deep neural network. Proceedings of the IEEE International Conference on Acoustic, Speech and Signal Processing.

[14] Liu Y., Qian Y., Chen N. (2015). Deep feature for text-dependent speaker verification. Speech Commun..

[15] Li N., Mak M., Chien J. Deep neural network driven mixture of PLDA for robust i-vector speaker verification. Proceedings of the IEEE Spoken Language Technology Workshop.

[16] Mitra V., Franco H. Time-frequency convolutional networks for robust speech recognition. Proceedings of the 2015 IEEE Workshop on Automatic Speech Recognition and Understanding (ASRU).

[17] Zhang Y., Pezeshki M., Brakel P., Zhang S., Bengio C.L.Y., Courville A. Towards end-to-end speech recognition with deep convolutional neural network. Proceedings of the Interspeech.

[18] Greenwood D.D. (1961). Critical bandwidth and the frequency coordinates of the basilar membrane. Acout. Soc. Am..

[19] Lepage E. (2003). The mammalian cochlear map is optimally warped. J. Acoust. Soc. Am..

[20] Carnero B., Drygajlo A. (1999). Perceptual speech coding and enhancement Using frame-synchronized fast wavelet packet transform algorithm. Trans. Signal Process..

[21] Almaadeed N., Aggoun A., Amira A. (2014). Speaker identification using multimodal neural network and wavelet analysis. Biometrics.

[22] Kenny P., Boulianne G., Dumouchel P. (2005). Eigenvoice Modeling with Sparse Trainning Data. IEEE Trans. Speech Audio Process..

[23] Wamg S., Qian Y., Yu K. What does the speaker embedding encode?. Proceedings of the Interspeech.

[24] George K.K., Kumar C.S., Ramachandran K.I., Ashish P. Cosine Distance Features for Robust Speaker Verification. Proceedings of the Interspeech.

[25] Klosowski P., Dustor A., Lzydorczyk J. (2015). Speaker verification performance evaluation based on open source speech processing software and TIMIT speech corpus. Comput. Netw..

[26] Nagrani A., Chung J.S., Zisserman A. VoxCeleb: A large-scale speaker identification dataset. Proceedings of the Interspeech.

[27] Daubechies I. (1988). Orthonormal basis of compactly supported wavelet. Comput. Pure Appl. Math..

[28] Yang Q., Wang J. (2015). Multi-level wavelet Shannon entropy-based method for signal-sensor sault location. Entropy.

[29] Daqrouq K. (2011). Wavelet entropy and neural network for text-independent speaker identification. Eng. Appl. Artif. Intell..

[30] Abdel-Hamid O., Mohamed A., Jiang H., Penn G. Applying convolutional neural network concepts to hybrid NN-HMM model for speech recognition. Proceedings of the International Conference on Acoustics, Speech and Signal Processing.

